# Sacral Fracture Causing Neurogenic Bladder: A Case Report

**DOI:** 10.1155/2012/587216

**Published:** 2012-02-14

**Authors:** Tatsuro Sasaji, Noboru Yamada, Kazuo Iwai

**Affiliations:** Department of Orthopedic Surgery, Fukushima Rosai Hospital, 3-Numajiri, Tsuzura-machi, Uchigo, Iwaki 973-8403, Japan

## Abstract

A 76-year-old man presented with a Denis Zone III sacral fracture after a traffic accident. He also developed urinary retention and perineal numbness. The patient was diagnosed with neurogenic bladder dysfunction caused by the sacral fracture. A computed tomogram (CT) revealed that third sacral lamina was fractured and displaced into the spinal canal, but vertebral body did not displace. The fracture lines began at the center of lamina and extended bilateraly. The fracture pattern was unique. The sacrum was osteoporosis, and this fracture may be based on osteoporosis. We performed laminectomy to decompress sacral nerve roots. One month after surgery, the patient was able to urinate. Three months after surgery, his bladder function recovered normally. One year after surgery, he returned to a normal daily life and had no complaints regarding urination. One-year postoperative CT showed the decompressed third sacrum without displacement.

## 1. Introduction

The sacrum is a large, triangular bone that connects the spine and pelvis through which sacral nerve roots run. Treatment of sacral fractures is based on the radiographic fracture pattern and neurological deficit. Denis and his colleagues classified sacral fractures into three types on the basis of the incidence of neurological injury [1]. Zone I fractures involve the ala of the sacrum to the lateral border of the neural foramen. Zone II fractures involve the neural foramen, and Zone III fractures involve the central portion of the sacrum. The incidence of neurological damage in patients with Zone III sacral fractures has been reported to be 56.7%, of which 76.1% patients developed bowel, bladder, and sexual dysfunctions. A series of sacral fractures have been reported till date, but case reports are few and details of sacral fracture remain unclear. Here, we report the details of radiological findings and therapeutic course for a patient with a Denis Zone III sacral fracture.

 The patient gave consent to submit data for publication.

## 2. Case Report

A 76-year-old man was hit from behind by a car and admitted to the hospital. He complained pain in the sacral area. After admission, he faced difficulty with urination and developed perineal numbness.

### 2.1. Neurological Examination

Neurological examination showed that his muscle power was normal in lower extremities and his ankle jerk decreased. Sensory disturbance was detected on the perineal region. His urinary retention was severe, sense of bladder urgency was lost, and defecation and bulbocavernosus reflex were normal. His urinary tract was not damaged. We diagnosed neurogenic bladder dysfunction. All laboratory findings were within normal limits.

### 2.2. Radiological Findings

**Figure 1:**
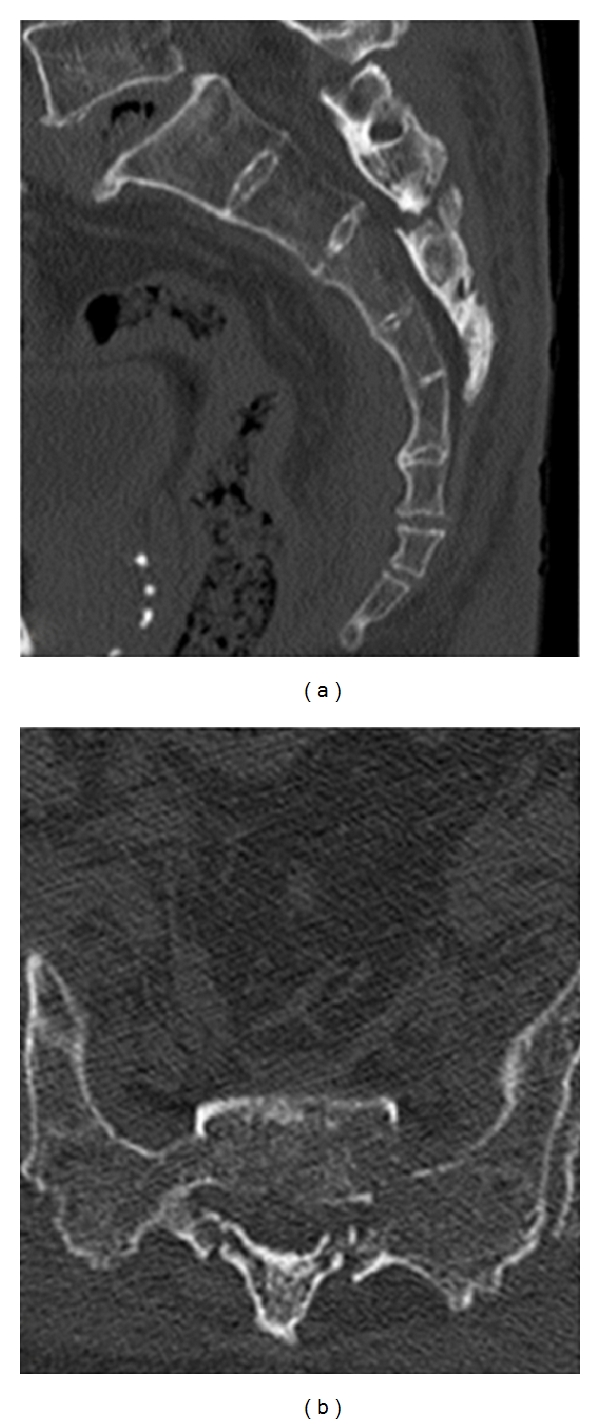
Preoperative reconstructed computed tomogram (CT). (a) Sagittal reconstructed CT: the third sacral lamina fractured and displaced into the spinal canal. The third sacral vertebral body did not angulate. (b) Axial reconstructed CT: the third sacral lamina fractured bilaterally. The spinal canal area decreased. The sacrum was osteoporosis.

**Figure 2:**
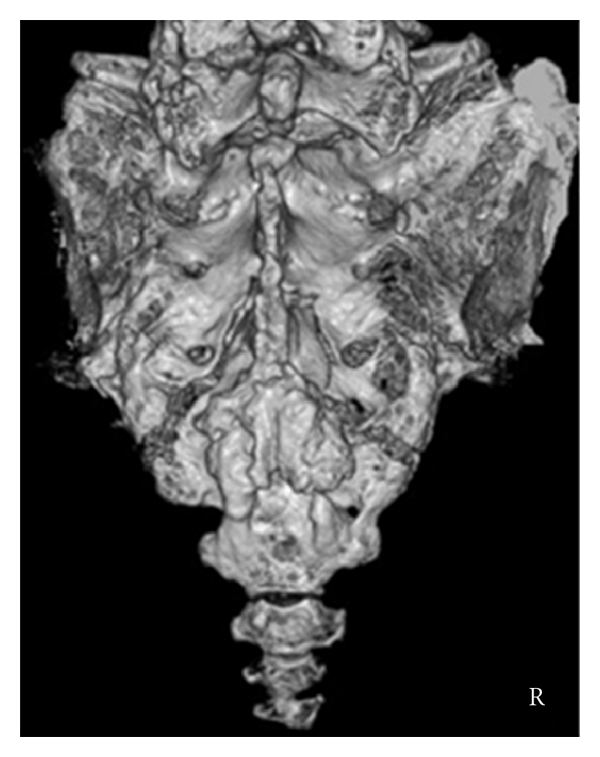
Preoperative three-dimensional CT: fracture lines began at the center of lamina and extended bilaterally.

**Figure 3:**
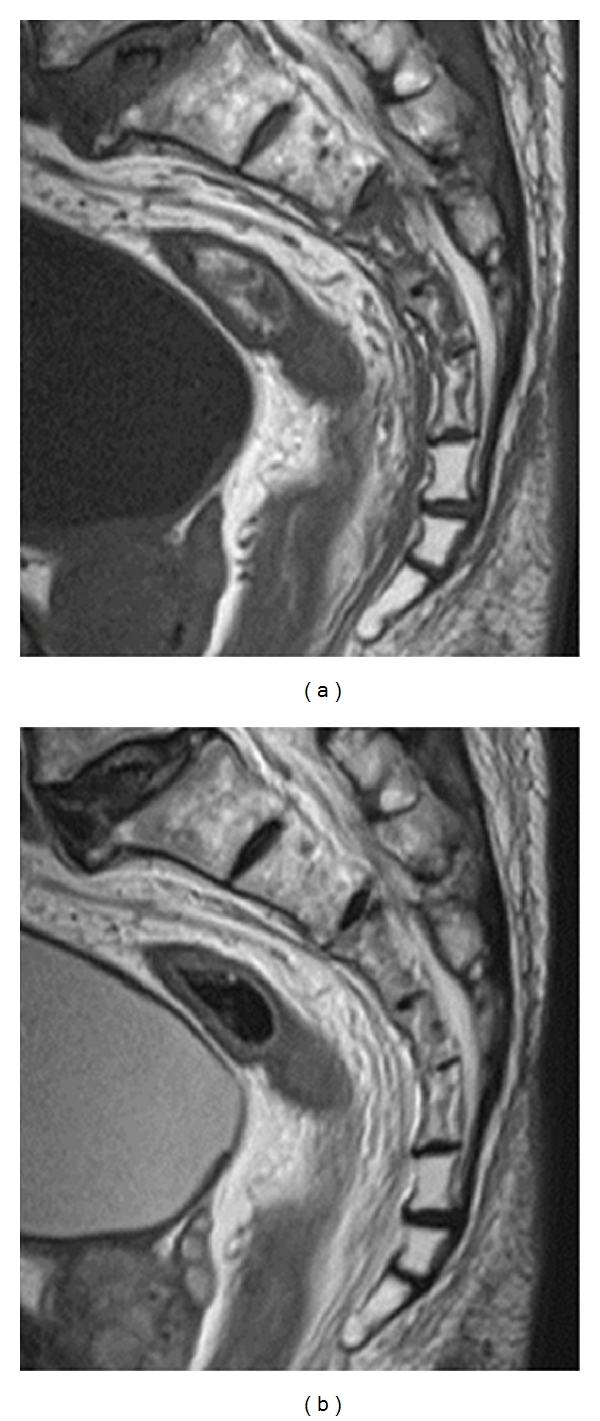
Preoperative magnetic resonance image. (a) Sagittal plane on a T1-weighted image and (b) sagittal plane on a T2-weighted image Third, fourth, and fifth sacral vertebral bodies showed low intensities on T1- and T2-weighted images.

A sagittal reconstructed computed tomogram (CT) revealed that the third sacral lamina was fractured and displaced into the spinal canal. The posterior part of the third sacral vertebral body became hollow, but the sacral vertebral body did not displace (Figure 1(a)). An axial reconstructed CT revealed that the third sacral lamina was fractured bilaterally. The cortex of sacrum was thin. The trabeculae of the third sacral body were sparse (Figure 1(b)). These appearance suggested osteoporosis. A three-dimensional CT revealed oblique fracture lines, which began at the center of the third sacral lamina and extended bilaterally (Figure 2). The fracture lines were unique, and we suspected the fragility fracture based on osteoprosis. The fracture involved Zone II and III. In relevance to clinical symptoms, we diagnosed Denis Zone III sacral fracture. The vertebral body did not displace, and so we determined that this fracture was stable. Our operation plan was a decompression surgery without stabilization procedure. Sagittal planes of magnetic resonance imaging showed signal changes in the sacral vertebral bodies. The third, fourth, and fifth sacral vertebral bodies showed low intensities on T1- and T2-weighted images (Figures 3(a) and 3(b)). We interpreted these findings as microfracture.

### 2.3. Operation

**Figure 4:**
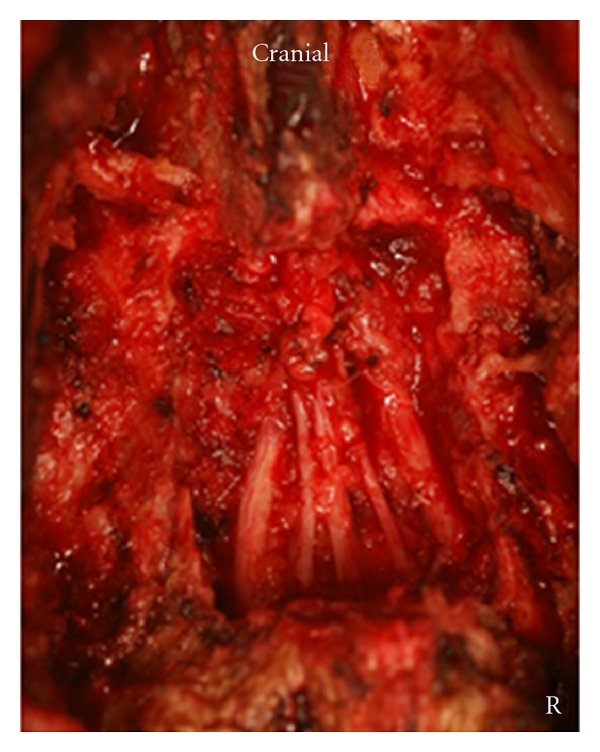
Intraoperative photograph: sacral nerve roots were decompressed.

The third sacral lamina was explored through a straight posterior midline approach. Laminectomy of the third sacral lamina was performed using a burr. No hematoma was observed. The sacral nerve roots were adhered and not disrupted (Figure 4).

### 2.4. Postoperative Course

**Figure 5:**
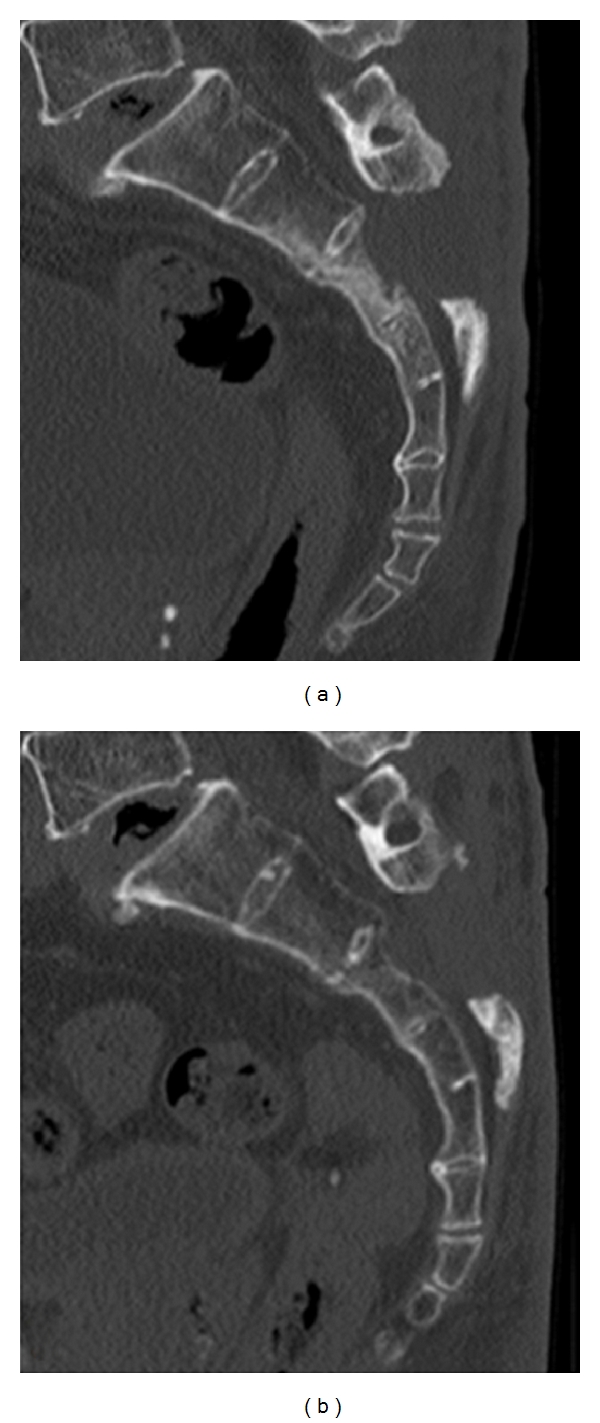
Postoperative follow-up reconstructed CT. (a) Two months after surgery, the third sacral vertebral body showed a high-density area. (b) One year after surgery, the third sacral vertebral body had jointed without displacement.

**Figure 6:**
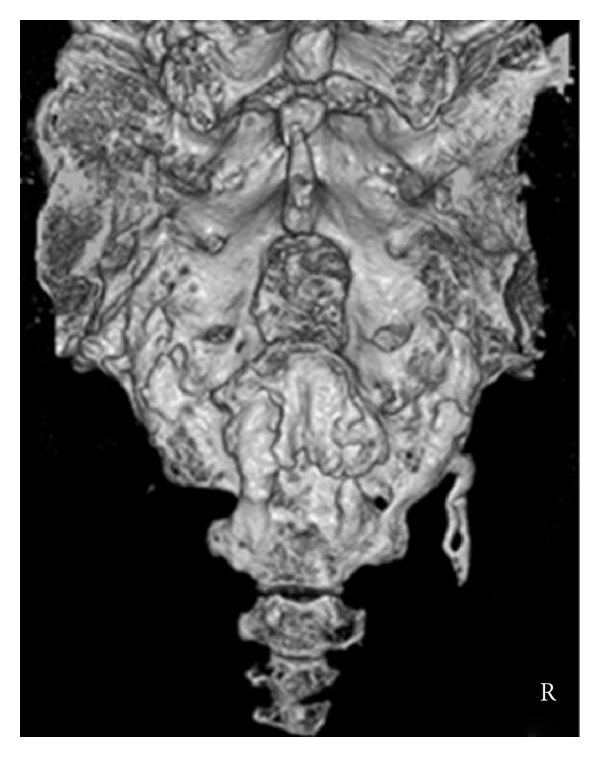
One-year postoperative follow-up three-dimensional CT Laminectomy remained and fracture lines in zone II disappeared.

A numbness around his penis improved soon after surgery. One month after surgery, he was able to urinate. Three months after surgery, his sensation of residual urine was lost and bladder function recovered normally. One year after surgery, he returned to a normal daily life, although perineal numbness remained. Two-month postoperative follow-up sagittal reconstructed CT showed high density area in third sacral vertebral body (Figure 5(a)). One-year postoperative follow-up sagittal reconstructed CT showed that the sacral vertebral body had jointed without displacement (Figure 5(b)). One-year postoperative follow-up three-dimensional CT revealed that laminectomy remained without displacement (Figure 6).

## 3. Discussion

There have been several reports on the incidence of sacral fractures associated with pelvic fractures. Denis reported 30.4%, Bonnin reported 45%, and Ueda reported 15.9% of such cases [1–3]. Because of few case reports on sacral fractures, the details of radiological findings and therapeutic course remain unclear [4–6].

 Sacral fracture patterns are difficult to understand by plain radiographs. According to a textbook about the spine, dedicated CT is useful for evaluation of sacral fractures; however, three-dimensional reformed CT add little additional insight [7]. In the present case, three-dimensional CT clearly revealed the fracture lines and we planed the operation on the basis of fracture pattern. Therefore, we recognized the usefulness of three-dimensional CT in the examination of sacral fractures.

According to Fisher, Gibbons, and Zelle, bladder function recovered completely in some cases of sacral fractures, although the time in which it recovered has not been mentioned in their reports [8–10]. In the present case, bladder function recovered completely in three months because the patient had a simple fracture. The time to recover bladder function in cases of severe sacral fracture may take more than three months.

The treatment of sacral fractures is based on fracture pattern and neurological status. There have been some reports that neurological status improved by both surgery and conservative treatment [1, 4, 9–13]. Treatment in cases of sacral fractures with neurological symptoms remains controversial. In the present case, a stable sacral fracture with neurological deficit was diagnosed. Therefore, we performed laminectomy without stabilization procedure, which resulted in a good neurological improvement.
